# A Novel Hybrid Precoding-Companding Technique for Peak-to-Average Power Ratio Reduction in 5G and beyond

**DOI:** 10.3390/s21041410

**Published:** 2021-02-18

**Authors:** Mohamed Mounir, Mohamed B. El_Mashade, Salah Berra, Gurjot Singh Gaba, Mehedi Masud

**Affiliations:** 1 Department of Electronics and Communications Engineering, El Gazeera High Institute for Engineering and Technology, Cairo 11751, Egypt; engmohamed.monir@gi.edu.eg; 2 Department of Electrical Engineering, Faculty of Engineering, Al-Azhar University, Cairo 11751, Egypt; mohamed.b.elmashade@azhar.edu.eg; 3 Department of Electronic and Telecommunications, Electrical Engineering Laboratory (LAGE), Kasdi Merbah University, BP 511, Ouargla 30000, Algeria; berra.salah@univ-ouargla.dz; 4 School of Electronics and Electrical Engineering, Lovely Professional University, Punjab 144411, India; gurjot.17023@lpu.co.in; 5 Department of Computer Science, College of Computers and Information Technology, Taif University, P.O. Box 11099, Taif 21944, Saudi Arabia

**Keywords:** 5G, beyond 5G (B5G), orthogonal frequency division multiplexing (OFDM), peak-to-average power ratio (PAPR) reduction, out-of-band (OOB) radiation reduction, high power amplifier (HPA), hybrid PAPR reduction techniques, precoding techniques, companding techniques, Partial Transmit Sequence (PTS)

## Abstract

Several high-speed wireless systems use Orthogonal Frequency Division Multiplexing (OFDM) due to its advantages. 5G has adopted OFDM and is expected to be considered beyond 5G (B5G). Meanwhile, OFDM has a high Peak-to-Average Power Ratio (PAPR) problem. Hybridization between two PAPR reduction techniques gains the two techniques’ advantages. Hybrid precoding-companding techniques are attractive as they require small computational complexity to achieve high PAPR reduction gain. Many precoding-companding techniques were introduced to increasing the PAPR reduction gain. However, reducing Bit Error Rate (BER) and out-of-band (OOB) radiation are more significant than increasing PAPR reduction gain. This paper proposes a new precoding-companding technique to better reduce the BER and OOB radiation than previous precoding-companding techniques. Results showed that the proposed technique outperforms all previous precoding-companding techniques in BER enhancement and OOB radiation reduction. The proposed technique reduces the Error Vector Magnitude (EVM) by 15 dB compared with 10 dB for the best previous technique. Additionally, the proposed technique increases high power amplifier efficiency (HPA) by 11.4%, while the best previous technique increased HPA efficiency by 9.8%. Moreover, our proposal achieves PAPR reduction gain better than the most known powerful PAPR reduction technique with a 99% reduction in required computational complexity.

## 1. Introduction

The high-speed data rate technologies such as 4G and 5G vastly use Orthogonal Frequency Division Multiplexing (OFDM) and expected to be used in Beyond 5G (B5G). Also, OFDM has been combined with most emerging communication techniques such as Cognitive Radio (CR), Massive Multiple-Input and Multiple-Output (mMIMO) [1,2]. However, OFDM has a high Peak-to-Average Power Ratio (PAPR) problem. This problem leads to excessive distortion in the OFDM signal due to the nonlinear High Power Amplifier (HPA) in the transmitter chain. Distortion in the OFDM signal causes degradation in the Bit Error Rate (BER) of the OFDM systems and grows the out-of-band (OOB) radiation. This problem can be solved by amplifying the signal with high PAPR with a large back-off or using a highly linear amplifier at the transmitter. The first solution causes HPA to work inefficiently, while the second solution is expensive. Thus, we need to reduce the PAPR of the OFDM signal [3].

Several PAPR reduction methods are introduced, such as clipping [4], Partial Transmit Sequence (PTS) [5], Selective Mapping (SLM) [6], Interleaving [7], Active Constellation Extension (ACE) [8], Tone Reservation (TR) [9], Tone Injection (TI) [9], companding techniques [10], and precoding techniques [11]. There are mainly three categories for PAPR reduction techniques: (i) coding techniques, (ii) Multiple Signal Representation (MSR) techniques, and (iii) adding-signal techniques [12]. Different metrics are used to compare PAPR reduction techniques, such as PAPR reduction gain, BER enhancement, OOB-radiation reduction, excess average power, rate loss, computational complexity, and downward compatibility. Generally, none of the PAPR reduction techniques can be claimed as the best PAPR reduction technique. Of course, each PAPR reduction technique has its advantages and disadvantages [3,13,14]. For example, MSR techniques have large PAPR reduction gain and well reduce BER and OOB-radiation. However, they require excessive computational complexity in addition to rate loss [15]. On the other hand, companding techniques have small PAPR reduction gain with trivial computational complexity [10]. Also, precoding techniques have moderate PAPR reduction gain with small computational complexity [11].

Hybridization between two or three PAPR reduction techniques is used to gain the advantages of the combined techniques. In the literature, different hybrid techniques combine different PAPR reduction techniques from different categories, or the same category [16]. For example, PTS-SLM [17], and PTS-Interleaving [18] are examples of hybridizing techniques that belong to the same category (i.e., MSR). On the other hand, ACE-PTS [19], precoding-clipping [20], PTS-companding [21], and precoding-companding [22] are examples of hybridizing different PAPR reduction techniques from different categories. Hybrid precoding-companding techniques are attractive as they require small computational complexity to achieve large PAPR reduction gain.

In the literature, many hybrid precoding-companding techniques exist. Walsh-Hadamard Transform-based precoding hybridized with μ-law (WHT-μ) [22] is the first precoding-companding technique introduced in the literature. In [23] WHT hybridized with exponential (exp) companding (WHT-exp) was compared with WHT-μ. However, the parameter of μ-law was not optimized to enhance its BER performance in [23].

The authors in [24] evaluated the performance of Discrete Hartley Transform hybridized technique with μ-law (DHT-μ) considering different companding profiles of μ-law. However, all the companding profiles showed the same performance in Rayleigh fading channel. The authors in [25] hybridized DHT with Piecewise Linear Companding (PLC). However, PLC is impractical. It requires many side information bits to inform the receiver with the position of data samples above or below the inflection point [10]. Recently, DHT-A introduced in [26] without comparison to any previous precoding-companding technique.

The authors in [27,28] showed that Discrete Fourier Transform hybridized with μ-law (DFT-μ) is better than DHT-μ from PAPR reduction and BER points of view. However, their model considered only the AWGN channel and neglected HPA. The authors in [29] introduced DFT-PLC. However, PLC is impractical, as discussed before.

The authors in [30] showed that DST-μ is better than the WHT-μ, DHT-μ, and DCT-μ from the PAPR reduction point of view. The model in [30] considered only the AWGN channel and neglected HPA. The authors in [31] introduced DST-A without any comparison with the previous precoding-companding techniques. The authors in [32] showed that DST-μ is better than DST-A and DCT-A from PAPR reduction and BER points of view. Recently, DCT-μ has been compared with DCT-μ-clipping in [33], only from the PAPR reduction point of view.

The authors in [34] showed that Zadoff–Chu Transform hybridized with μ-law (ZCT-μ) is better than WHT-μ from PAPR reduction point of view. The authors in [35] hybridized ZCT with the Piecewise Exponential Companding technique. However, the solution is impractical because it uses inflection point and side information data as PLC. Finally, Square root Raised Cosine-based precoding was hybridized with μ-law (SRC-μ) in [36,37] without compared to the previous precoding-companding techniques.

The limitations of the discussed works are summarized as follows:

- The authors in [22,24,27,28,30,32,33,34,36,37] hybridized μ-law with different precoding matrices due to its high PAPR reduction gain. However, BER and OOB radiation are more important metrics than PAPR reduction gain when working with PAPR reduction techniques that cause BER degradation, such as companding techniques.
- Some works hybridized A-law companding [26,31] and exp companding [23] with different precoding matrices. Although A-law and exp companding techniques have PAPR reduction gain and BER performance worse than the μ-law companding, as shown in [10].
- The authors in [25,29] hybridized piecewise based companding techniques with different precoding matrices. However, piecewise based companding techniques are impractical.
- Many precoding-companding techniques [26,29,31,36,37], are not compared with any previous works.
- HPA was neglected in the models [22,26,27,28,30,31,33,34,35,36,37], although HPA is the source of nonlinear distortion.
- The authors in [22,26,28,30,31,34,36,37] have not considered Rayleigh channel. However, OFDM was designed originally for Rayleigh channels.
- Most importantly, the previous works did not clarify why the precoding matrices are selected, and the companding transforms are hybridized. Although this is the key challenge in the hybridization process.

This paper proposes a new hybrid precoding-companding technique to reduce the BER and OOB radiation in the presence of HPA hybridizing Log companding and SRC precoding. The Log companding is selected because it is better than other practical companding techniques in terms of BER enhancement and OOB radiation reduction [10].

Also, the SRC precoding outperforms all other techniques [11]. Hence, hybrid SRC-Log can better reduce the BER and OOB radiation than the previous precoding-companding techniques. The proposed SRC-Log is compared with the ten state of the art precoding-companding techniques (i.e., DHT-*A* [26], SRC-μ [37], DCT-μ [33], DFT-μ [28], DST-μ [32], DHT-μ [24], WHT-μ [22], WHT-exp [23], DST-*A* [31], and ZCT-μ [34]) in the presence of HPA from PAPR reduction gain, OOB radiation, Error Vector Magnitude (EVM), and BER points of view.

### Contributions

- This paper proposes a new precoding-companding technique to reduce BER and OOB radiation.
- The proposed technique outperforms state of the art precoding-companding techniques in terms of BER, EVM, and OOB-radiation reduction.
- The proposed technique reduces EVM by 15 dB and increases HPA efficiency by 11.4% in contrast with the best-known technique (i.e., SRC-μ) that reduces EVM by 10 dB and increases HPA efficiency by 9.88%.
- The proposed technique also achieves PAPR reduction gain better than PTS, the most powerful PAPR reduction technique with a 99% reduction in the required computational complexity.

The paper is organized as follows: Section 2 discusses the proposed technique’s background and problem description. Section 2.1 highlights the importance of PAPR reduction in OFDM systems. Section 2.2 describes criteria for PAPR reduction techniques. Section 3 presents the proposed technique for PAPR reduction (i.e., SRC-Log), and simulation results are presented in Section 4. Finally, Section 5 concludes the paper.

*Notations:* Scalars are represented by small italic letters. Small and capital letters refer to time and frequency domains, respectively. Matrices are represented by bold normal letters. Subscript *n* and *k* refer to the time sample and subcarrier index, while superscript *c* and *e* denote companded and expanded signals, respectively. Variable *t* and *f* denote the time instant and frequency component in the continuous domain, respectively. Operators |·|, ∠·, $\hat{\cdot }$, E{·}, and $Re\left\{\cdot \right\}$ denotes absolute, angle, estimated value, average value, and real part of complex value, respectively. P{·} is used to denote probability density functions (PDF). Finally, $j=\sqrt{-}1$.

## 2. Background and Motivation

OFDM is the most popular multicarrier modulation technique. Multicarrier is used in both 4G and 5G systems. It is also expected that B5G will consider OFDM. OFDM reduces the computational complexity of the equalization process than the adaptive equalizer. It simply converts the multipath channel into a flat channel over each subcarrier. Thus, a simple one-tap equalizer is required for each subcarrier. In the OFDM transmitter, the incoming modulated symbols (e.g., QPSK or M-QAM) are grouped into $N_{T}$ parallel symbols and go through the $N_{T}$-point IFFT. Equation (1) represents the time domain OFDM symbol [14];

(1) $$x_{n}=\frac{1}{\sqrt{2}}\sum_{k=0}^{N_{T}-1}A_{k}e^{j\frac{2\pi nk}{N_{T}}},\;\;0\leq n\leq N_{T}-1$$

where $N_{T}$ denotes the total subcarriers’ number of the OFDM symbol and $A_{k}$ represents the modulating symbol of the $k_{th}$ frequency domain subcarrier. After the IFFT process, a cyclic prefix (CP) is added to convert the multipath channel into a circular convolutional channel to simplify the equalization process in the transmitter [38]. Let $a_{n}$ and $b_{n}$ be the real and imaginary parts of $x_{n}$ (i.e., $x_{n}=a_{n}+jb_{n}$). For a sufficiently large number of subcarriers (usually, N≥64) $a_{n}$ and $b_{n}$ will fit the Gaussian distribution in accordance with the central limit theorem (CLT). Thus, envelope of OFDM symbol (i.e., $|x_{n}|=\sqrt{a_{n}+b_{n}}$) will follow a Rayleigh distribution, while its power follows the chi-squared distribution with degree of freedom equal two. The chi-squared distribution indicates that the OFDM symbol’s maximum power is very large with respect to the average power of the OFDM symbol (i.e., large PAPR). Mathematical representation of the PAPR of the discrete-time baseband OFDM symbol is expressed as follows [14]:

(2) $$PAPR=\frac{\max_{n\in \left[0,N_{T}\right]}{\left|x_{n}\right|}^{2}}{E\left\{|x_{n}{|}^{2}\right\}}$$

where E{.} refers to the arithmetic mean. Continuous-time passband OFDM symbol has PAPR value greater than the PAPR of the OFDM symbol in baseband by 3 dB [3,13,14]. The passband OFDM signal is expressed as [3]

(3) $$s\left(t\right)=Re\left\{x\left(t\right)e^{j2\pi f_{c}t}\right\}$$

where

(4) $$x\left(t\right)=\frac{1}{\sqrt{2}}\sum_{k=0}^{N_{T}-1}A_{k}e^{j\frac{2\pi kt}{N_{T}T_{s}}},\;\;0\leq t\leq T_{OFDM}$$

$T_{OFDM}=T_{s}+T_{g}$ where $T_{s}$ is the duration of the OFDM symbol and $T_{g}$ is the duration of CP. If the input to the HPA is $s\left(t\right)=\left|s\left(t\right)\right|e^{j∠s\left(t\right)}$, where $|s\left(t\right)|$ and $∠s\left(t\right)$ are the amplituded and phase angle of $s\left(t\right)$ respectively, then the output of the memory-less nonlinear HPA is $y\left(t\right)=\Lambda \left[|s\left(t\right)|\right]e^{j\{∠s\left(t\right)+\Theta \left[|s\left(t\right)|\right]\}}$, where $\Lambda \left[\cdot \right]$ and $\Theta \left[\cdot \right]$ represent the amplitude/amplitude (AM/AM) and amplitude/phase (AM/PM) conversions of HPA, respectively (i.e., $\Lambda \left[\cdot \right]$ and $\Theta \left[\cdot \right]$ describe the effect of non-linearity on $|s\left(t\right)|$ and $∠s\left(t\right)$, resperctivelly) [13,38].

In the receiver, AWGN is added to the received signal from the channel. The received baseband signal $\hat{x}\left(t\right)$ is then converted to digital form $r_{n}$. Then, CP is removed from each OFDM symbol before being converted to the FFT process’s frequency domain. Frequency domain OFDM symbol is expressed as [38]:

(5) $${\hat{A}}_{k}=\frac{1}{\sqrt{2}}\sum_{k=0}^{N_{T}-1}r_{n}e^{j\frac{2\pi nk}{N_{T}}},\;\;0\leq k\leq N_{T}-1$$

Finally, equalized OFDM symbol (i.e., ${\hat{A}}_{k}/H_{k}$) is de-mapped before serialization, where $H_{k}$ is the complex channel gain on subcarrier $k_{th}$ [38].

### 2.1. Motivation for PAPR Reduction

**Figure 1:**
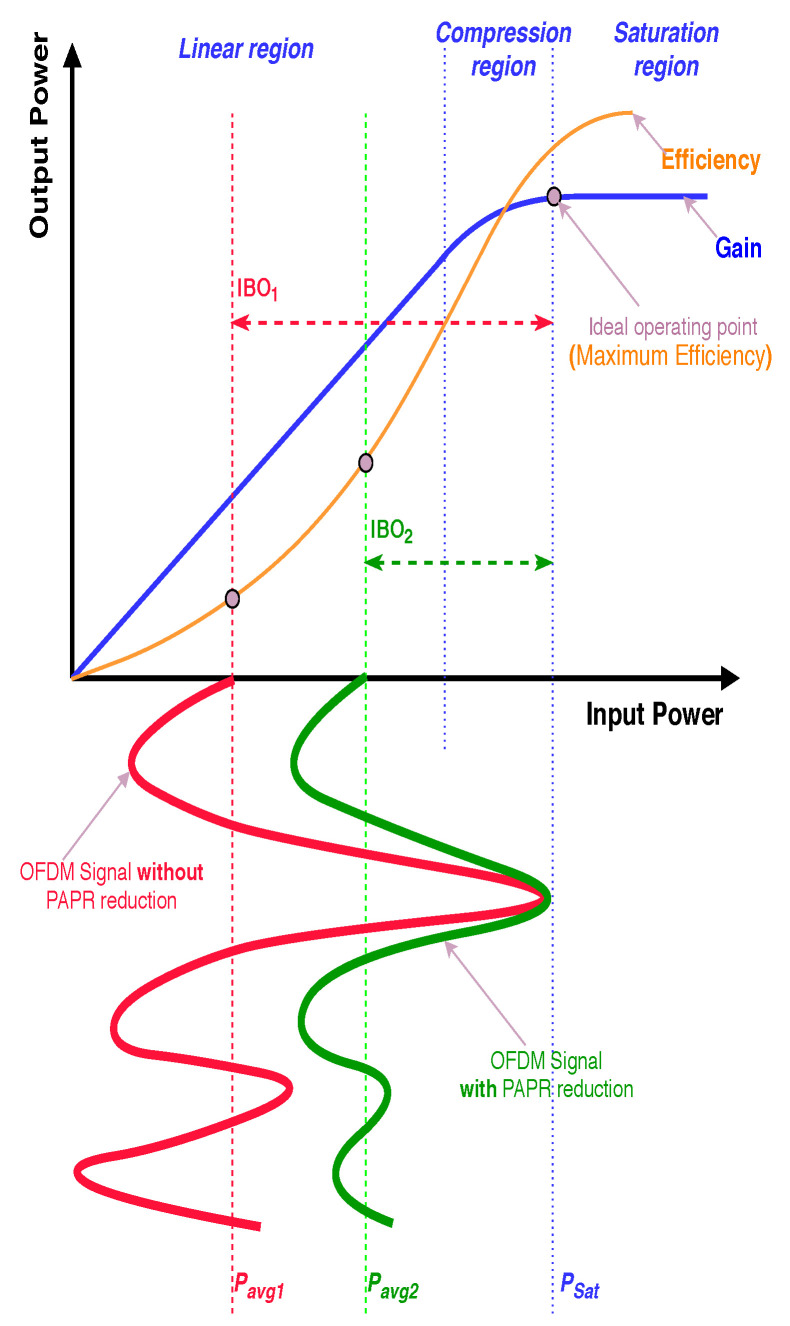
General AM/AM characteristic of HPA.

This section emphasizes the importance of PAPR reduction. Because HPA is the primary source of nonlinear distortion in OFDM-based systems, an adequate HPA model must be considered. Different models exist for memoryless HPAs, such as Rapp model, Ghorbani model, and Saleh model [38]. Generally, AM/AM characteristics of any memoryless HPA have three regions, namely linear region, compression region, and saturation region, as illustrated in Figure 1. Nonlinear distortion severity relies on the value of the input back-off (IBO), which is given by [16];

(6) $$IBO=10\log_{10}\left(\frac{P_{sat}}{P_{avg}}\right)=10\log_{10}\left(\frac{s_{sat}^{2}}{E\left\{|s\left(t\right){|}^{2}\right\}}\right)$$

Or

(7) $$IBO=P_{sat}\;\;\left[dB\right]-P_{avg}\;\;\left[dB\right]$$

where $P_{sat}$ is the saturation level of input power, $P_{avg}$ is the input signal average power, and $s_{sat}$ is the saturation level. Predistortion is usually used to convert the compression region into a linear region to reduce the required back-off. AM/AM characteristics of linearized HPA (also called soft Limiter (SL)) is expressed as [3];

(8) $$\Lambda \left[|s\left(t\right)|\right]=\left\{\begin{array}{cc} s(t) & \left|s\left(t\right)\right|\leq s_{sat}\\ s_{sat}e^{-j∠s(t)} & |s\left(t\right)|>s_{sat} \end{array}\right.$$

Theoretically, IBO must be equal to the PAPR of an input signal (i.e., IBO = PAPR) to avoid nonlinear distortion. However, this degrades the HPA efficiency, which is related to the back-off and PAPR as follows [13,16];

(9) $$\eta =\frac{\eta_{max}}{IBO}=\frac{\eta_{max}}{OBO}=\frac{\eta_{max}}{PAPR}$$

where $\eta_{max}$ is the maximum efficiency depending on the HPA class, $\eta_{max}=0.5$ for class A and 0.785 for class B. It is worth mentioning that in the case of linearized HPA (i.e., SL), output back-off (OBO) is equal to IBO. Due to this inverse relationship between linearity and HPA efficiency, we have to reduce the PAPR of the OFDM signal [13]. Figure 1 shows that HPA efficiency has its maximum value at the saturation level. In this figure, the original OFDM signal (red) has to work with high IBO ($IBO_{1}$) to avoid distortion. Modified OFDM signal (green) after PAPR reduction requires low IBO ($IBO_{2}$). There are different selection criteria exits to choose from among the several PAPR reduction techniques. The following section discusses different selection criteria.

### 2.2. Evaluation Criteria of PAPR Reduction Techniques

There are different metrics used in the comparison of different PAPR reduction techniques. The main three metrics of them are; PAPR reduction gain, BER enhancement, and OOB-radiation reduction. PAPR reduction gain is the most famous metric at all. However, PAPR is a statistical quantity. Therefore, the probability of the OFDM symbol’s PAPR is larger than or equal to a threshold $\xi_{o}$ is described by aid of Complementary Cumulative Distribution Function (CCDF), which is expressed as:

(10) $$CCDF=P\{PAPR\geq \xi_{o}\}$$

Theoretical CCDF of the PAPR of the original oversampled OFDM signal is given by [13,16];

(11) $$CCDF_{c}=1-exp\left(\sqrt[{-N_{T}e^{-\xi_{o}}}]{\frac{\pi }{3}\log N_{T}}\right)$$

Oversampling by factor L (usually L = 4 is enough) is used in the simulation of continuous-time signal’s PAPR [3,13,14].

In contrast to CCDF, BER and OOB-radiation are affected by the HPA characteristics. EVM is another metric similar to BER, which is usually used when Forward Error Correction (FEC) is not considered. EVM evaluates the in-band distortion. The mathematical definition of EVM is represented as follows [3,38]:

(12) $$EVM=\sqrt{\frac{E\left\{|{\hat{A}}_{k}-A_{k}{|}^{2}\right\}}{E\left\{|A_{k}{|}^{2}\right\}}}$$

Reduction in OOB-radiation may be measured by Adjacent Channel Power Ratio (ACPR), which is defined as adjacent Channel’s power(out-of-band distortion) to the main Channel’s power (in-band signal)ratio. ACPR is defined as follows [38]:

(13) $$ACPR=10\log\left(\left(\int_{F_{out}}Y\left(f\right)df\right)/\left(\int_{F_{in}}Y\left(f\right)df\right)\right)$$

where $F_{in}$ and $F_{out}$ are defined as the limits of inband and outband, respectively. $Y\left(f\right)$ is defined as the power spectral density (PSD) of HPA output (i.e., $y\left(t\right)$).

Another important metric to be considered is the computational complexity. Computational Complexity Reduction Ratio (CCRR) between two techniques is formulated as follows [39];

(14) $$\mathrm{CCRR}=\frac{\mathrm{CC}\mathrm{of}\mathrm{the}\mathrm{conventional}\mathrm{technique}-\mathrm{CC}\mathrm{of}\mathrm{the}\mathrm{proposed}\mathrm{technique}}{\mathrm{CC}\mathrm{of}\mathrm{the}\mathrm{conventional}\mathrm{technique}}\times 100\%$$

*CCRR* is computed for both numbers of additions and multiplications.

## 3. Proposed Hybrid Precoding-Companding Technique

This research aims to propose a competitive PAPR reduction technique for low latency applications in 5G and B5G, such as autonomous driving and vehicle to vehicle communications. Techniques with minimal computational complexity are favorable for low latency applications. Considering the computational complexity, hybrid precoding-companding techniques are the first selection for PAPR reduction. Unfortunately, precoding-companding techniques degrade the BER performance and increase OBB-radiation in the presence of HPA and may worsen them than the case of no PAPR reduction technique is used. Lliterature review reveals that previous precoding-companding techniques are concerned in the PAPR reduction gain increase. Most of the previous works did not explain the rationale of selection among different precoding matrices and different companding transforms to be used in hybridization. In other words, they did not mention how and why they choose the precoding matrix or the companding transform used.

We propose a precoding-companding technique to enhance the BER and reduce the OBB-radiation instead of increasing the PAPR reduction gain. The technique selects the best precoding and companding techniques from BER and OOB-radiation reduction points of view.

**Figure 2:**
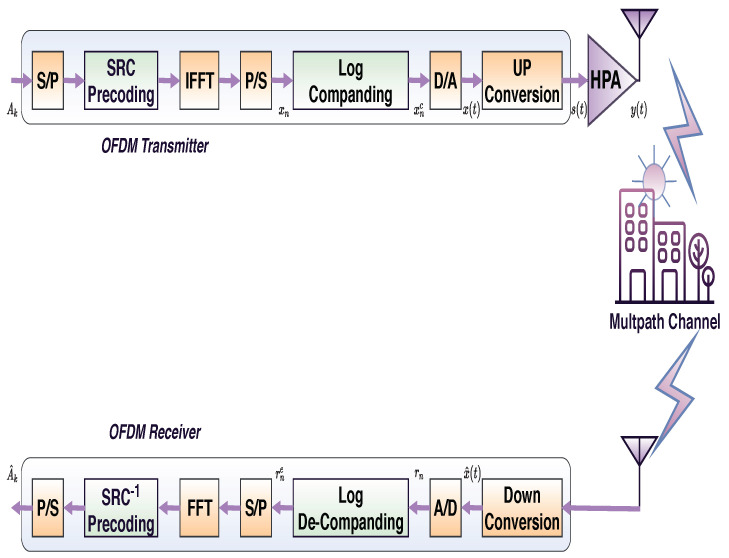
General block diagram of SRC-Log-based OFDM system.

The authors in [11,10] showed that SRC precoding (Section 3.1) and Log companding (Section 3.2) are the best precoding and the best companding techniques from BER and OOB-radiation reduction points of view. Therefore, they are selected to be hybridized in the proposed technique, and their parameters are adjusted to increase the reduction of BER and OOB-radiation. Figure 2 shows the block diagram of the proposed model. The model considers HPA into account, Rayleigh and AWGN channels are simulated. In contrast, most of the previous works neglect HPA and simulate only the AWGN channel. However, OFDM was designed originally for Rayleigh channels, and PAPR reduction techniques are used to mitigate the nonlinear distortion of HPA.

Although the proposed technique’s goal is to decrease BER and OBB-radiation; this does not mean that Although the PAPR reduction gain of the proposed technique is limited. Although To highlight the proposed technique’s PAPR reduction capability, the proposed technique is compared with PTS (The most powerful PAPR reduction technique that has excessive computational complexity) from PAPR reduction and computational complexity points of view. The computational complexity of the proposed technique is the summation of SRC precoding and Log companding. In (17) and (19), respectively, the computational complexity of both SRC precoding and Log companding is determined. They are not calculated in the previous works.

### 3.1. Precoding

Precoding techniques achieve large PAPR reduction gain with small computational complexity. It does not require Side information or distort the transmitted signal. However, most of the precoding matrices lead to more distortion in nonlinearity than the original signal. Fortunately, the SRC matrix does not increase the distortion. However, it causes some data rate loss [11]. Precoding matrix **R** is given as:

(15) $$R=\left[\begin{array}{cccc} R_{1,1} & R_{1,2} & \cdots & R_{1,\left(N_{T}-N_{R}\right)}\\ R_{2,1} & R_{2,2} & \cdots & R_{2,\left(N_{T}-N_{R}\right)}\\ \vdots & \vdots & \ddots & \vdots \\ R_{N_{T},1} & R_{N_{T},2} & \cdots & R_{N_{T},\left(N_{T}-N_{R}\right)} \end{array}\right]$$

SRC precoding matrix spreads $\left(N_{T}-N_{R}\right)$ subcarriers on $N_{T}$ subcarriers, where $0\leq N_{R}<N_{T}$ is the loss in terms of subcarriers. Losses in subcarriers become zero if $N_{R}$ equals zero, this causes SRC matrix to be DFT matrix. Values of $R_{n,m}$ are defined as follows [40]:

(16) $$R_{n,m}=R_{n,o}e^{j\left(\frac{2\pi nm}{N_{T}}\right)}$$

where $R_{n,o}=\left\{\begin{array}{cc} \frac{{\left(-1\right)}^{n}}{\sqrt{2}}\sin\left(\frac{\pi n}{2N_{R}}\right), & 0\leq n\leq N_{R}\\ \frac{{\left(-1\right)}^{n}}{\sqrt{2}}, & N_{R}\leq n\leq \left(N_{T}-N_{R}\right)\\ \frac{{\left(-1\right)}^{n}}{\sqrt{2}}\cos\left(\frac{\pi (n-N_{T})}{2N_{R}}\right), & \left(N_{T}-N_{R}\right)\leq n\leq N_{T} \end{array}\right.$

The computational complexity of the SRC precoding is characterized by the number of real multiplications ($RM_{s}$) and the number of real additions ($RA_{s}$) which are given as follows:

(17a) $$RM_{s}=4N_{DSC}\left(N_{DSC}-N_{R}\right)$$

(17b) $$RA_{s}=2N_{DSC}\left(2\left(N_{DSC}-N_{R}\right)-1\right)$$

where $N_{DSC}$ is the number of data subcarriers.

### 3.2. Companding

Companding is a promising technique for PAPR reduction, and it has a moderate PAPR reduction gain with the smallest computational complexity among all other PAPR reduction techniques. However, the companding technique achieves PAPR reduction at the expense of degrading BER performance. There exist different companding transforms. For each companding transform, there is a point at which BER degradation is the minimum. It is the efficient operating point, regardless of PAPR reduction gain [41]. Among all companding transforms, Log companding with a threshold is the best in BER performance and OOB radiation reduction. BER and OOB radiation reduction are more important than PAPR reduction gain in companding techniques. Transfer functions of Log companding and de-companding with threshold are defined as follows [10]:

(18a) $$x_{n}^{c}=\left\{\begin{array}{cc} x_{n} & ,|x_{n}|\leq x_{th}\\ a\ln\{1+\left(x_{n}b\right)\} & ,|x_{n}|>x_{th} \end{array}\right.$$

(18b) $$r_{n}^{e}=\left\{\begin{array}{cc} r_{n} & ,|r_{n}|\leq x_{th}\\ (exp^{\;\left(r_{n}/a\right)}-1)/b & ,|r_{n}|>x_{th} \end{array}\right.$$

Log companding compresses signals with an amplitude larger than $x_{th}$ while keeping signals with small amplitude unchanged. Two parameters a and b controlling Log transform slope (a×b). The slope of the transform must equal to unity (a×b=1), to keep OFDM symbol average power the same before and after the transform [3].

This research assumes companding with filtering to alleviate OOB radiation, although filtering may produce some peak regrowth [10]. Frequency domain filtering consists of two IFFT processes. The first FFT process converts the OFDM signal into a frequency domain. Then the in-band frequency domain components of the companded signal $x_{o}\ldots x_{\left(N_{DSC}/2-1\right)},x_{\left(LN_{DSC}-\left(N_{DSC}/2+1\right)\right)}\ldots x_{\left(LN_{DSC}-1\right)}$ are kept as it is, while OOB components of the companded signal $x_{\left(N_{DSC}/2\right)}\ldots x_{\left(LN_{DSC}-N_{DSC}/2\right)}$ are nulled. The second IFFT process converts the signal back to the time domain. Frequency domain filtering does not require a reverse process in the receiver [42]. In the transmitter, computational complexity in terms of $RM_{s}$ and $RA_{s}$ of the Log companding transform is given by:

(19a) $$RM_{s}=\left(i_{Taylor}+1\right)6N_{T}$$

(19b) $$RA_{s}=\left(4i_{Taylor}+1\right)N_{T}$$

where $i_{Taylor}$ is the number of terms in Taylor series that can be sufficiently set to 10.

## 4. Simulation and Results

This section discusses the performance results of the proposed SRC-Log and the comparison results with the ten state of the art precoding-companding techniques (i.e., DHT-*A* [26], SRC-μ [37], DCT-μ [33], DFT-μ [28], DST-μ [32], DHT-μ [24], WHT-μ [22], WHT-exp [23], DST-*A* [31], and ZCT-μ [34]) in terms of PAPR reduction gain, BER, OOB radiation reduction, and EVM. Moreover, the PAPR reduction performance of SRC-Log is compared with PTS. Parameters of the simulation are given in Table 1.

**Figure 3:**
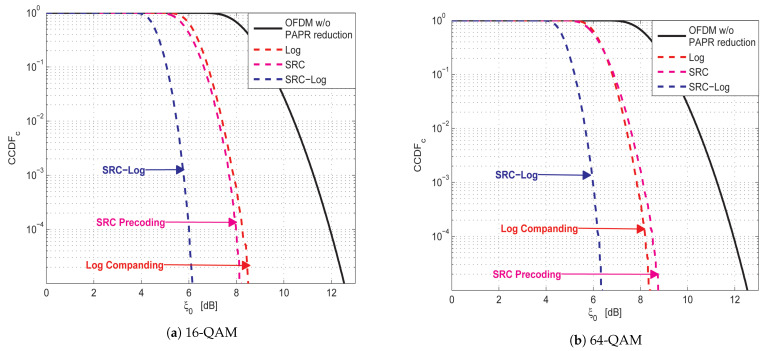
PAPR reduction capability comparison between proposed SRC-Log technique and conventional SRC precoding and Log companding techniques.

**Figure 4:**
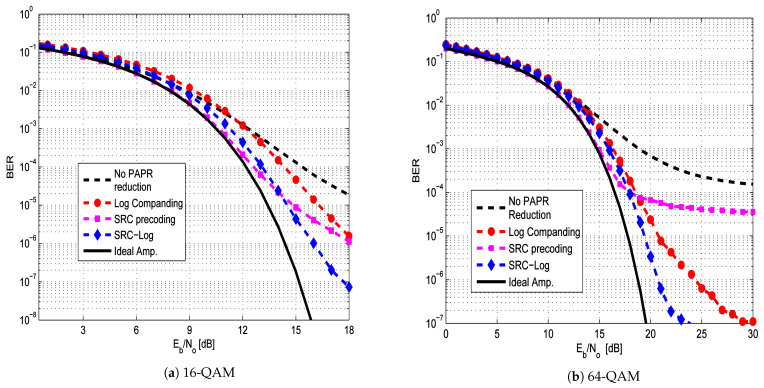
BER performance of SRC-Log in AWGN channel in comparison with SRC precoding and Log companding, along with OFDM w/o PAPR reduction. The case of an ideal amplifier is shown in a solid line.

**Figure 5:**
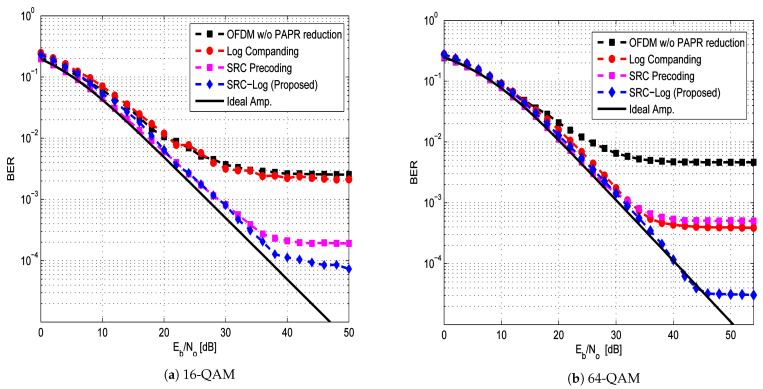
BER performance of SRC-Log in Rayleigh channel in comparison with SRC precoding and Log companding, along with OFDM w/o PAPR reduction. The case of an ideal amplifier is shown in a solid line.

Firstly, the PAPR reduction performance of SRC-Log is compared with the conventional SRC precoding and conventional Log companding, as shown in Figure 3. Figure 4 and Figure 5 show the comparative performance of SRC-Log with conventional SRC precoding and conventional Log companding in AWGN channel and Rayleigh channel, respectively. As expected, SRC-Log has better PAPR reduction and BER performance than SRC precoding and Log companding. However, extra computational complexity is the price of performance enhancements, as shown in Table 2 that is based on (17) and (19).

It is observed in 16-QAM (Figure 4a and Figure 5a) that SRC precoding has better BER performance than Log companding, while in 64-QAM (Figure 4b and Figure 5b) SRC precoding has worse BER performance than Log companding. This is because precoding techniques have PAPR reduction performance depends on modulation order. This can be observed in Figure 3, where SRC precoding has better PAPR reduction performance than Log companding in 16-QAM (Figure 3a) and worse than in 64-QAM (Figure 3b).

**Figure 6:**
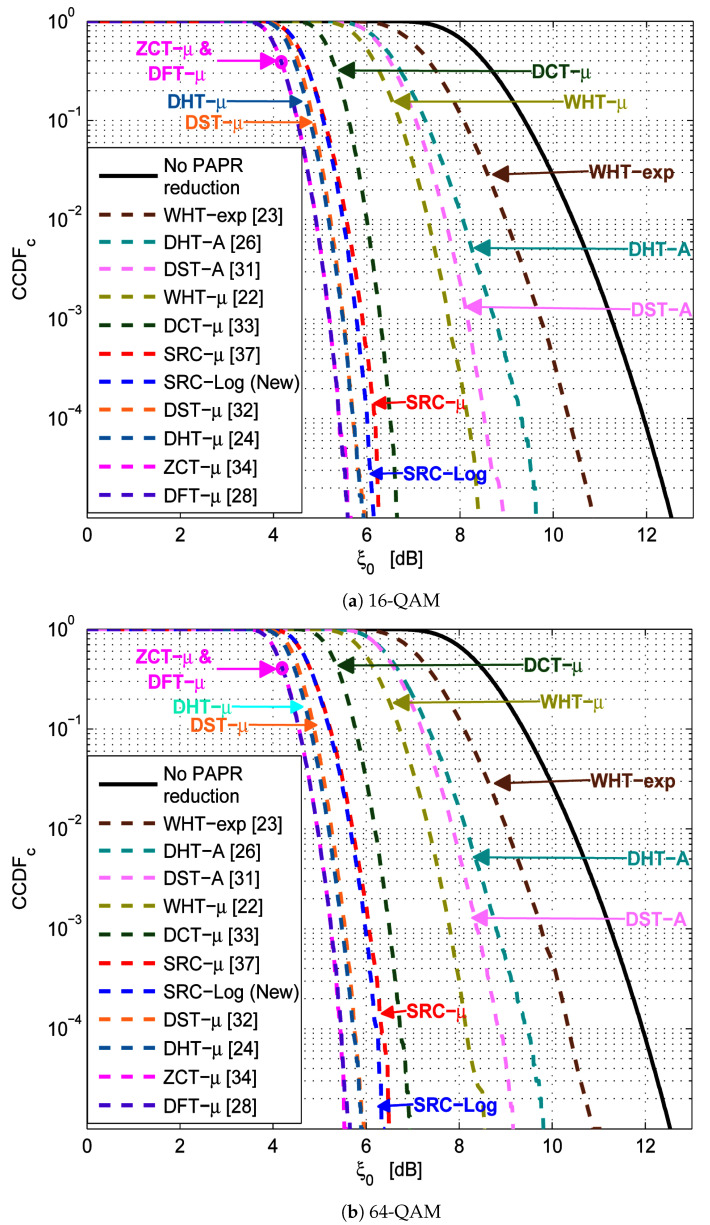
PAPR reduction capability of the new SRC-Log technique and the previous precoding-companding techniques.

Figure 6 shows the comparison results of the PAPR reduction capability of SRC-Log with the ten state of the art precoding-companding techniques (i.e., DHT-*A* [26], SRC-μ [37], DCT-μ [33], DFT-μ [28], DST-μ [32], DHT-μ [24], WHT-μ [22], WHT-exp [23], DST-*A* [31], and ZCT-μ [34]). As shown in Figure 6a,b, in 16-QAM and 64-QAM PAPR reduction performance is almost similar. Clearly, in 16-QAM and 64-QAM DHT-μ, DST-μ, and WHT-μ are better than DHT-*A*, DST-*A*, and WHT-exp. This is because μ-law companding has PAPR reduction performance better than *A*-law companding and exp companding [10]. Not surprisingly, ZCT-μ, DFT-μ, DHT-μ, and DST-μ have PAPR reduction performance better than SRC-Log. As, μ-law companding has PAPR reduction performance better than Log companding, especially at low PAPR values. However, at high PAPR values, Log becomes hardly better than μ-law. Although ZCT-μ, DFT-μ, DHT-μ, and DST-μ have PAPR reduction performance better than SRC-Log, this does not mean they are better than SRC-Log because PAPR reduction performance is not the primary metric to determine the best technique. Especially when working with the techniques that cause BER degradation as a PAPR reduction cost, such as companding techniques.

**Figure 7:**
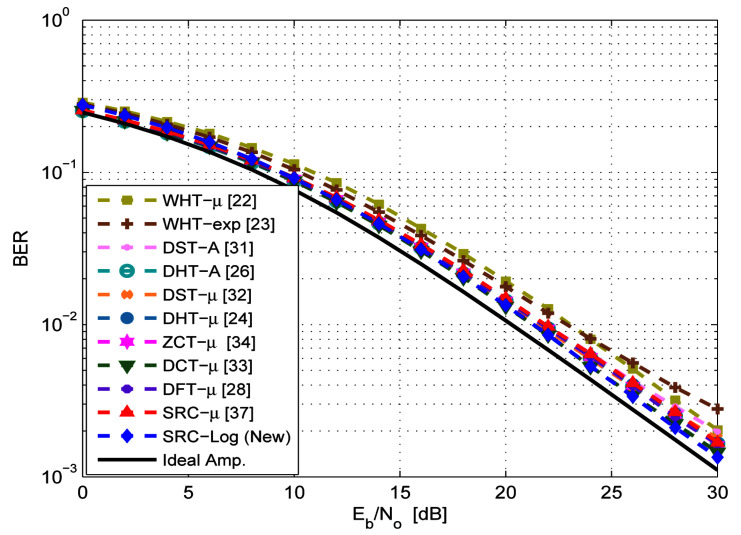
BER performance of the new SRC-Log techniques in Comparison with the previous precoding-companding techniques in the linear amplifier cases. In addition to the case of OFDM w/o PAPR reduction (solid line).

**Figure 8:**
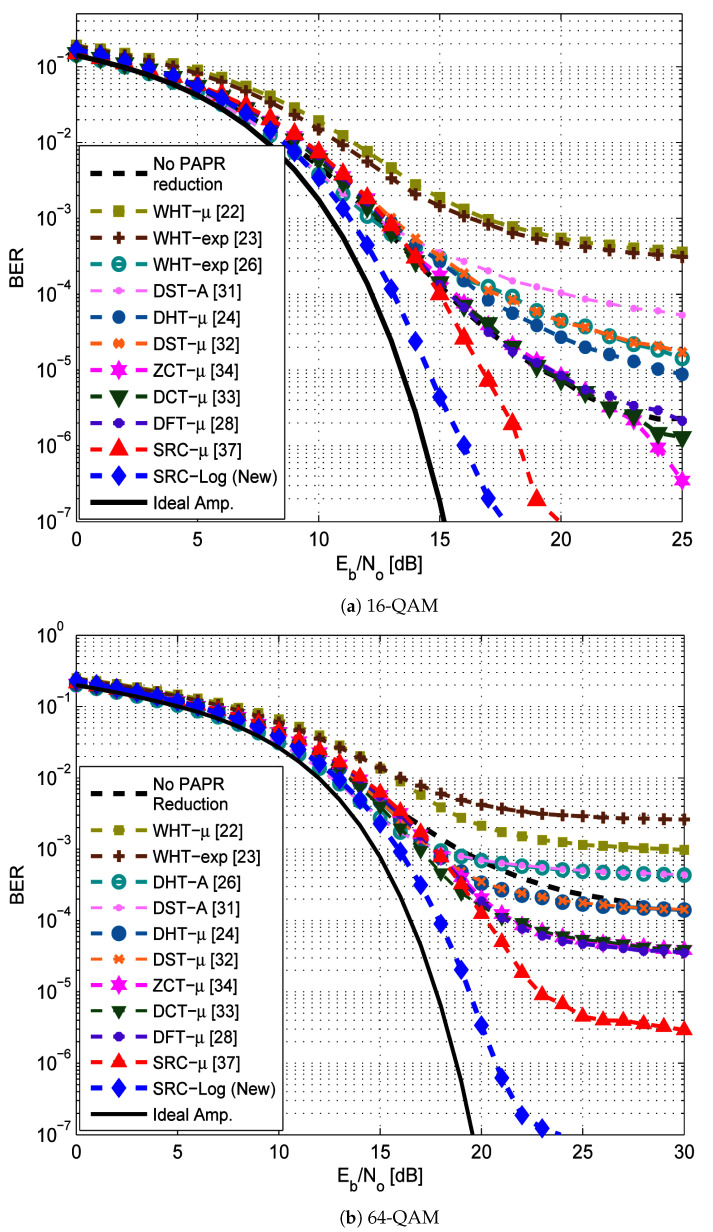
BER performance of the new SRC-Log compared with the previous precoding-companding techniques and OFDM without PAPR reduction in AWGN channel. In addition to the case of an ideal amplifier (solid line).

**Figure 9:**
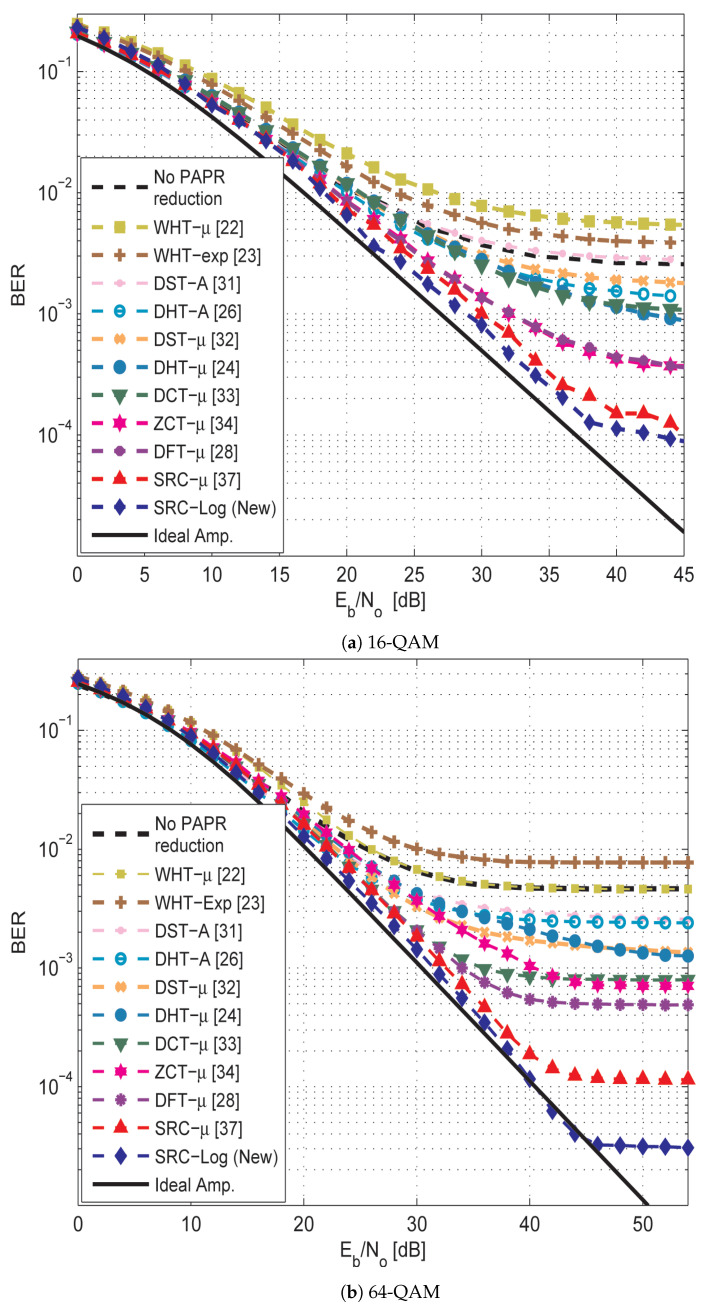
BER performance of the new SRC-Log compared with the previous precoding-companding techniques and OFDM without PAPR reduction in Rayleigh channel. In addition to the case of an ideal amplifier (solid line).

Both μ-law and Log companding causes BER degradation due to the receiver’s channel noise expansion. Figure 7 shows the BER degradation of the SRC-Log technique and the previous techniques under the ideal amplifier. The previous techniques and the proposed technique show small BER degradation as a cost of PAPR reduction. The BER degradation performance of each one of them is slightly different based on the associated precoding matrix. The proposed SRC-Log has the lowest BER degradation, among others. Although they all have similar BER degradation performance in the presence of an ideal (linear) amplifier, their BER performance are highly different under the non-ideal (practical) amplifier, as shown in Figure 8 and Figure 9.

**Figure 10:**
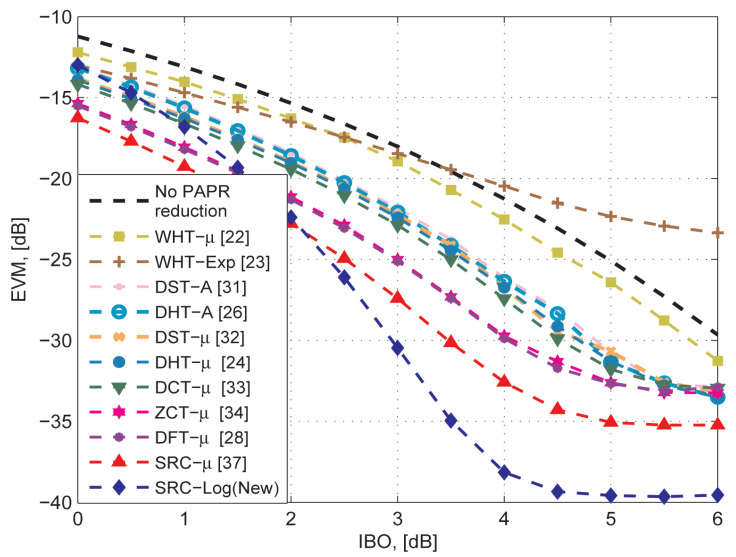
EVM comparison of the proposed SRC-Log and the previous precoding- companding techniques, in addition to the cases of OFDM w/o PAPR reduction in the presence of nonlinearity.

Figure 8 compares the BER performance of the SRC-Log technique with the ten state-of-art precoding-companding techniques in the presence of the non-ideal amplifier (HPA) with the AWGN channel. Figure 9 compares them in the Rayleigh channel. It is observed that SRC-Log has the best BER performance than the other techniques. Figure 10 demonstrates this and shows comparison results EVM versus IBO for the precoding-companding techniques at a given SNR in the Rayleigh channel. It is observed that for the same IBO (especially IBO ≥ 2 dB), SRC-Log has EVM lower than the other precoding-companding techniques. For instance, SRC-Log reduces the EVM by 10 dB at IBO = 5 dB compared to 5 dB reduction by SRC-mu (the best previous technique). However, at low IBO (impractical choice), Log companding is more distorted than the μ-law companding.

In Figure 8 and Figure 9, interestingly note that some precoding-companding techniques (e.g., WHT-μ and WHT-exp) have BER performance worse than or hardly equal to the performance of OFDM without PAPR reduction techniques. This is due to the orthogonality of their associated precoding matrices become more distorted by nonlinearity noise than IFFT. Also, some precoding-companding techniques which have the same PAPR reduction performance (e.g., DST-μ and DHT-μ), have different BER performance due to the different capabilities of precoding techniques and companding techniques in mitigating the nonlinearity distortion.

**Figure 11:**
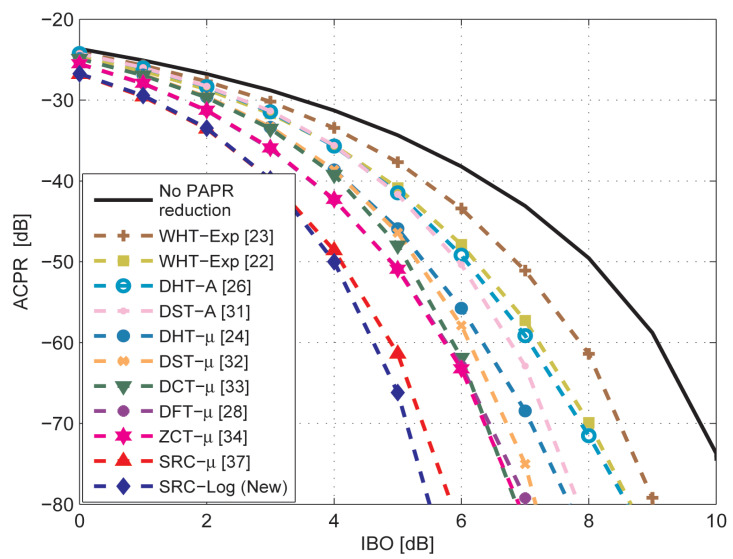
ACPR comparison of the proposed SRC-Log and the previous precoding- companding techniques, in addition to the cases of OFDM w/o PAPR reduction in the presence of nonlinearity.

OOB radiation reduction of different PAPR reduction techniques is usually compared using ACPR. Figure 11 compares ACPR versus IBO for the precoding-companding techniques at a given SNR. Obviously, for the same IBO (especially high IBO), SRC-Log has lower ACPR than the other precoding-companding techniques. For example, if −70 dB ACPR is required, then the OFDM system has to work with IBO = 9.09 dB without any PAPR reduction technique. Hence, HPA efficiency η is 6.29% ($\eta_{max}=50\%$). However, if SRC-μ (the best previous technique) is used, then the required IBO reduces to 4.9 dB, and efficiency η increases to 16.1%. On the other hand, if SRC-Log is used, then the required IBO reduces to 4.5 dB and efficiency η increases to 17.77%. It implies that SRC-Log increases the HPA efficiency η by 11.4% compared with 9.8% for the best previous technique (SRC-μ).

**Figure 12:**
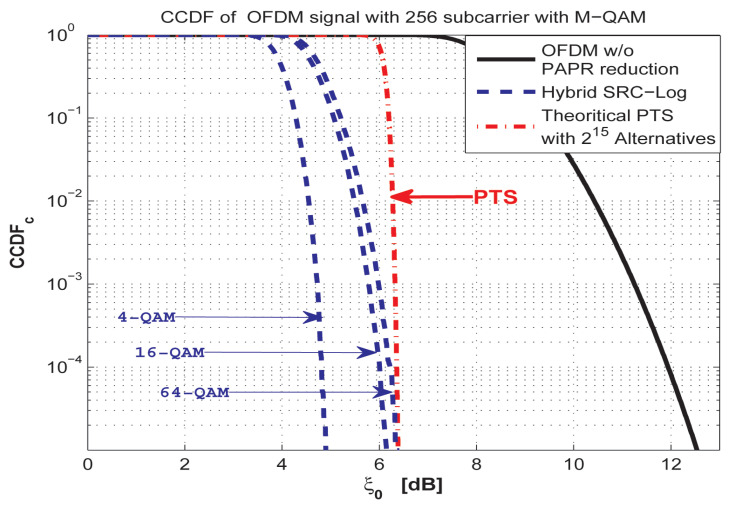
Comparison between SRC-Log and PTS with 215 alternatives. In the case of 4, 16, and 64-QAM.

**Figure 13:**
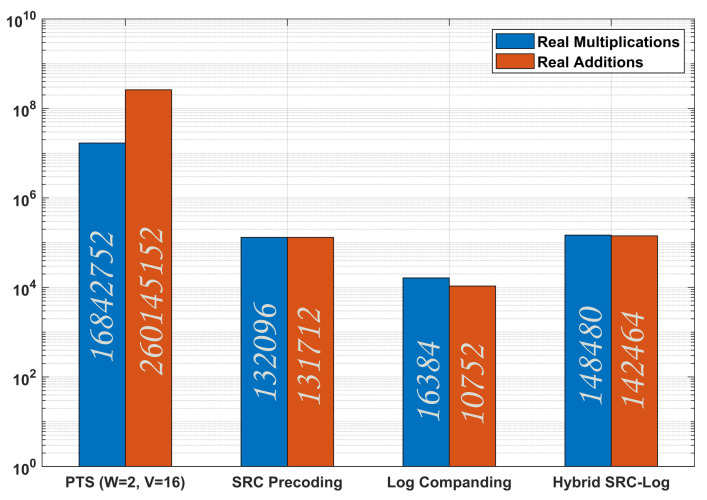
Comparison of computational complexity of SRC, Log, and Hybrid SRC-Log, based on (17), (19), along with PTS [15] with V = 16 and W = 2.

As shown in Figure 12, SRC-Log has better PAPR reduction performance than the Partial Transmit Sequence (PTS) in the case of 4, 16, and 64-QAM. The PAPR reduction gain comes with CCRR equal to 99% to PTS for both RAs and RMs, as shown in Figure 13 based on Table 2. This makes SRC-Log a useful technique for PAPR reduction.

From the above discussion, it can be concluded that the proposed SRC-Log is the best practical precoding-companding technique from different points of view. Although the proposed technique (SRC-Log) introduces better performance with small computational complexity compared with other techniques; its performance is limited in very small IBO values (e.g., IBO < 2 dB) as all PAPR reduction techniques. It means that HPA does not work with maximum efficiency, and power loss occurs at low IBO values. Recently, some works tried to reduce the PAPR by controlling the power allocated per subcarrier. Power allocation [43,44] and PAPR reduction can be jointly used to optimize the PAPR in OFDM systems.

## 5. Conclusions

Hybrid precoding-companding techniques are attractive ones. They provide high PAPR reduction gain with small computational complexity. Previous precoding-companding techniques are designed to increase PAPR reduction gain. Here, we propose a precoding-companding technique to reduce the BER and OOB better than previous ones, as this is the goal of PAPR reduction. Results showed that SCR-Log achieves OOB radiation reduction and BER performance better than the previous precoding-companding techniques. So, SCR-Log can be considered as the best precoding-companding technique. Moreover, SCR-Log achieves PAPR reduction performance better than PTS, with 99% CCRR to PTS. In the end, SCR-Log is an attractive precoding-companding technique to be used in OFDM systems. In the future, SCR-Log can be integrated with power allocation strategies to attain high-level PAPR optimization.

## Figures and Tables

**Table 1:** List of simulation parameters.

Parameters	Values
No. Subcarriers ($N_{T}$)	256
No. Data subcarriers ($N_{DSC}$)	192
Oversampling value (L)	4
Model of HPA	SL
Modulation (order-type)	16-QAM 64-QAM
Channel model	AWGN channel Rayleigh channel
IBO (dB)	16-QAM: 4/2 (AWGN/Rayleigh) 64-QAM: 5/4 (AWGN/Rayleigh)
Channel Estimation	Ideal
Decoder Type	Hard Decision Decoding

**Table 2:** Computational complexity comparison of SRC, Log, SRC-Log and PTS [15], based on (17) and (19).

Technique	No. of RMs	No. of RAs
SRC	132,096	131,712
Log	16,384	10,752
SRC-Log	148,480	142,464
PTS [15]	16,842,752	260,145,152

## Abbreviations

The following abbreviations are used in this manuscript:

ACE	Active Constellation Extension	MSR	Multiple Signal Representation
ACPR	Adjacent Channel Power Ratio	OBO	Output Back-Off
AM	Amplitude Modulation	OFDM	Orthogonal Frequency Division Multiplexing
AWGN	Additive White Gaussian Noise	OOB	Out-of-Band
BER	Bit Error Rate	PAPR	Peak-to-Average Power Ratio
B5G	Beyond 5G	PDF	Probability Density Functions
CCDF	Complementary Cumulative Distribution Function	PLC	Piecewise Linear Companding
CCRR	Computational Complexity Reduction Ratio	PM	Phase Modulation
CLT	Central Limit Theorem	PSD	Power Spectral Density
CP	Cyclic Prefix	PTS	Partial Transmit Sequence
CR	Cognitive Radio	RAs	Real Additions
DCT	Discrete Cosine Transform	RMs	Real Multiplications
DFT	Discrete Fourier Transform	SL	Soft Limiter
DHT	Discrete Hartley Transform	SLM	Selective Mapping
DST	Discrete Sine Transform	SRC	Square root Raised Cosine
EVM	Error Vector Magnitude	TI	Tone Injection
FEC	Forward Error Correction	TR	Tone Reservation
HPA	High Power Amplifier	VLC	Visible Light Communication
IBO	Input Back-Off	WHT	Walsh-Hadamard Transform
IFFT	Inverse Fast Fourier Transform	ZCT	Zadoff–Chu Transform
mMIMO	massive Multiple-Input and Multiple-Output	
